# Supplementary material for: Intronic CNVs and gene expression variation in human populations
Source: PLoS Genet. 2019 Jan 24;15(1):e1007902. doi: 10.1371/journal.pgen.1007902 (PMC6345438; doi:10.1371/journal.pgen.1007902)
Supplement: S1 Fig — Only variants in autosomes are considered and private events are excluded. (A) Number and type of CNVs per dataset. (B) Autosomal Mb that are CNV. Gray part of the bars corresponds to the CNV Mbs that are shared among maps. Colored parts of the bars are map-specific CNV regions. (C) Width distribution of gains and losses in each map. Bean lines and overall line are means). (D) Number of subjects and number of populations of origin used for building of each filtered map. DGV: Database of Genomic Variants (http://dgv.tcag.ca). For more information on the 1000 Genomes Project, see http://www.internationalgenome.org. (PDF) [file pgen.1007902.s001.pdf]

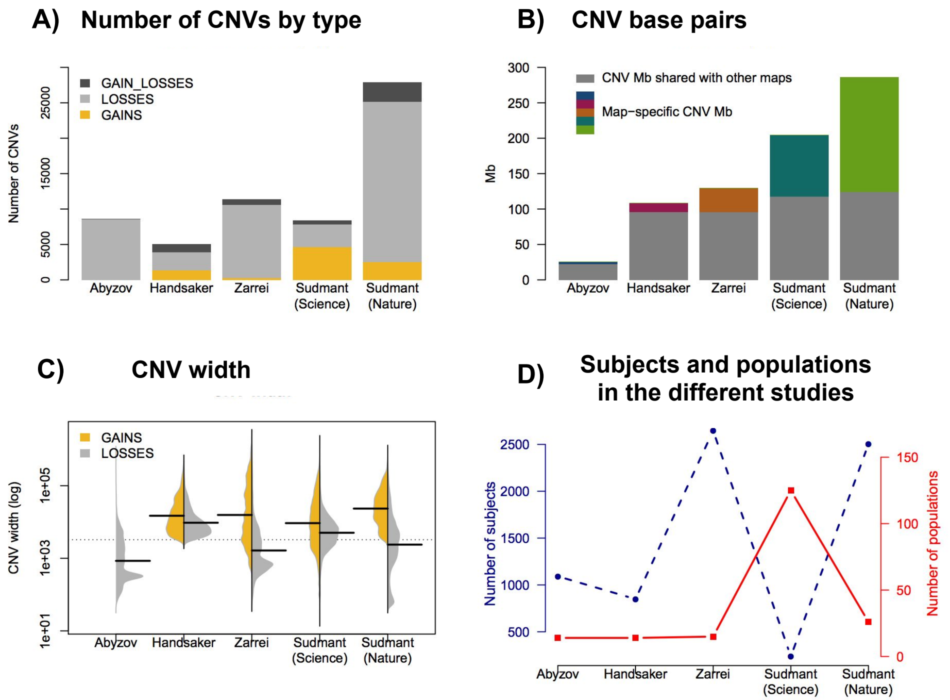

A) Number of CNVs by type
GAIN_LOSSES
LOSSES
GAINS
Number of CNVs
0
5000
15000
25000
Abyzov
Handsaker
Zarrei
Sudmant (Science)
Sudmant (Nature)
B) CNV base pairs
CNV Mb shared with other maps
Map−specific CNV Mb
Mb
0
50
100
150
200
250
300
Abyzov
Handsaker
Zarrei
Sudmant (Science)
Sudmant (Nature)
C) CNV width
GAINS
LOSSES
CNV width (log)
1e+01
1e+03
1e+05
Abyzov
Handsaker
Zarrei
Sudmant (Science)
Sudmant (Nature)
D) Subjects and populations in the different studies
Number of subjects
500
1000
1500
2000
2500
Number of populations
0
50
100
150
Abyzov
Handsaker
Zarrei
Sudmant (Science)
Sudmant (Nature)
