# Supplementary figures and images for: Intronic CNVs and gene expression variation in human populations
Source: PLoS Genet. 2019 Jan 24;15(1):e1007902. doi: 10.1371/journal.pgen.1007902 (PMC6345438; doi:10.1371/journal.pgen.1007902)

**A) Global background model**

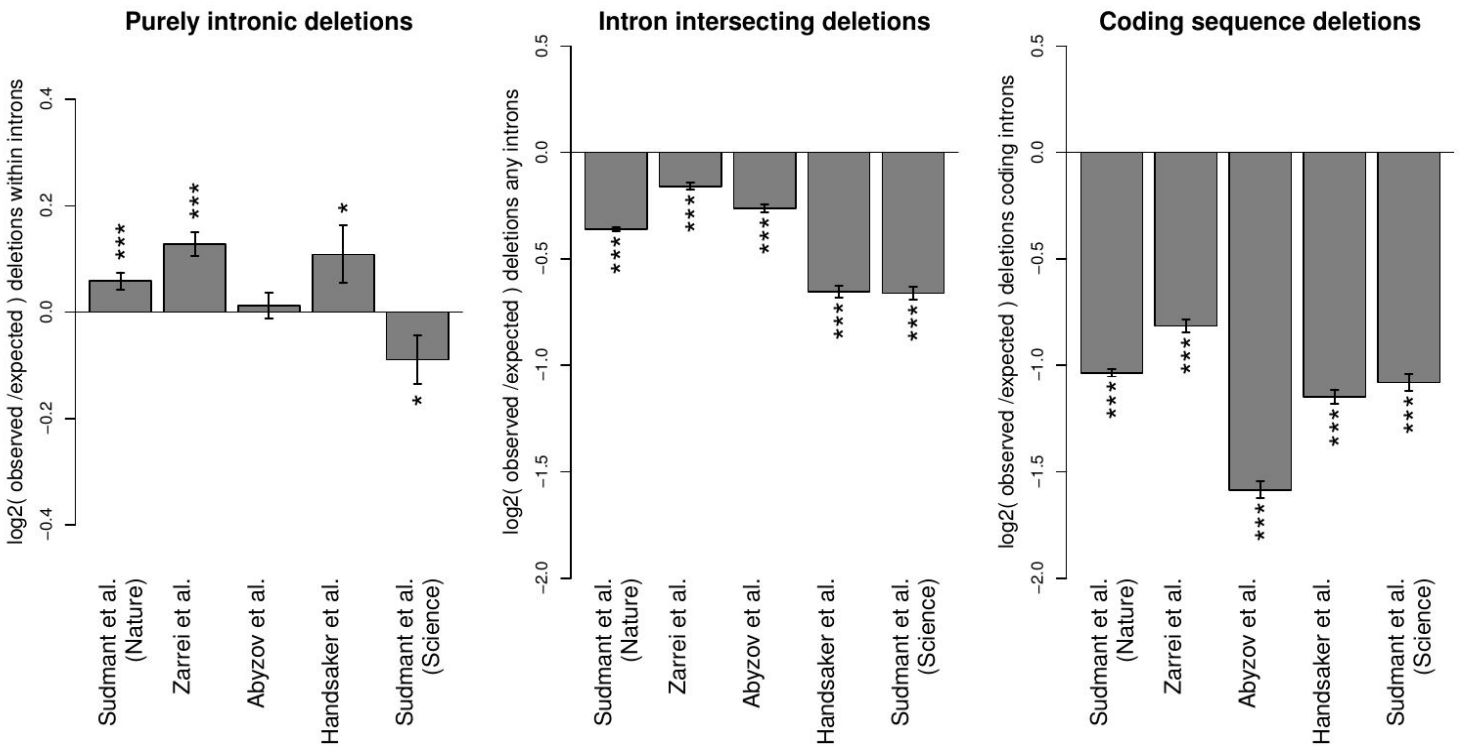

**B) Local background model**

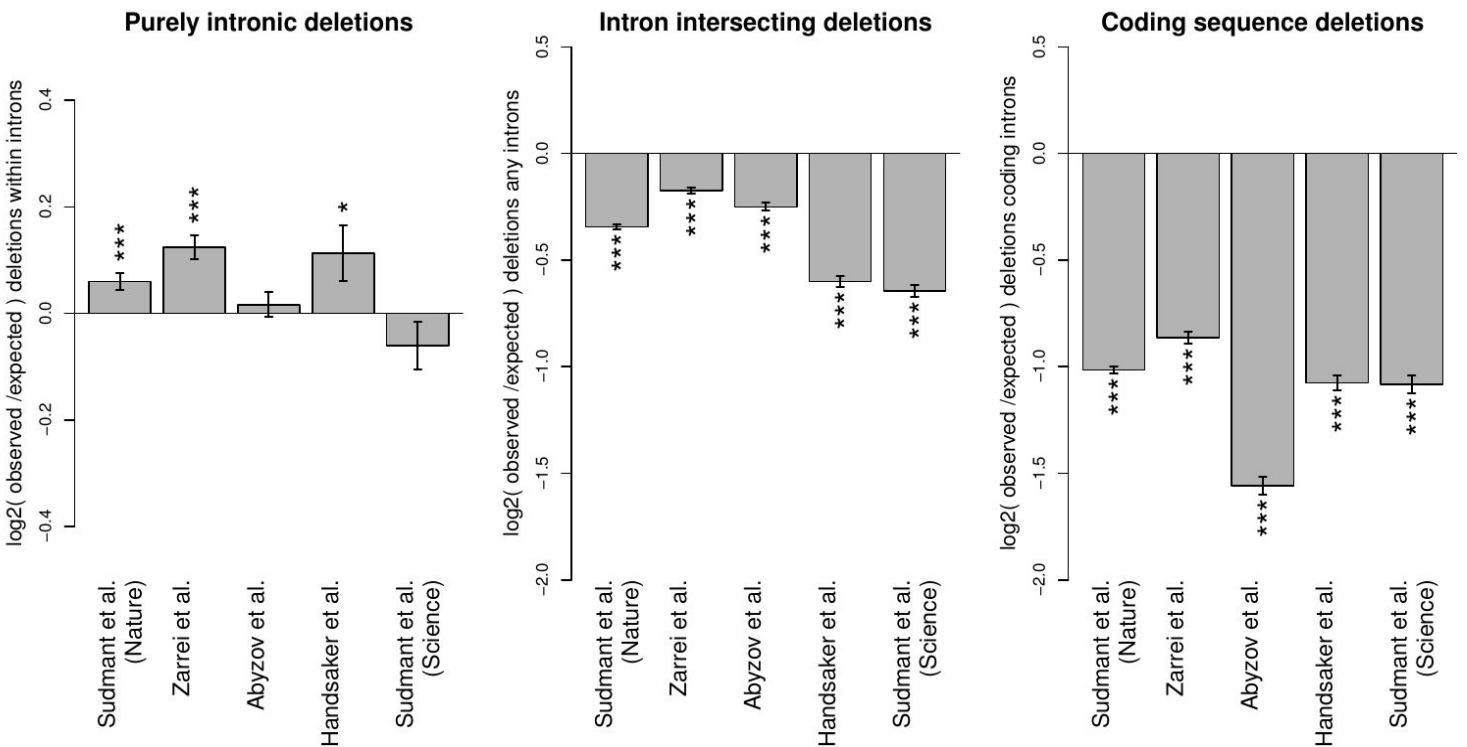

Supplement: S2 Fig — Enrichment or impoverishment of deletions within introns, deletions intersecting introns (purely intronic and intron-exon combined) and exon-overlapping deletions (purely coding and intron-exon combined) in different maps of copy number variation and using global (A) and local (B) background models. Values are given as log2 ratios observed/expected (median expected value from 10,000 randomisations). Error bars show the median absolute deviation and asterisks indicate significance: * for P<0.05, ** for P<0.005 and *** for P<0.0005. (PDF) [file pgen.1007902.s002.pdf]

# Comparison of deletion content in intronic and intergenic regions

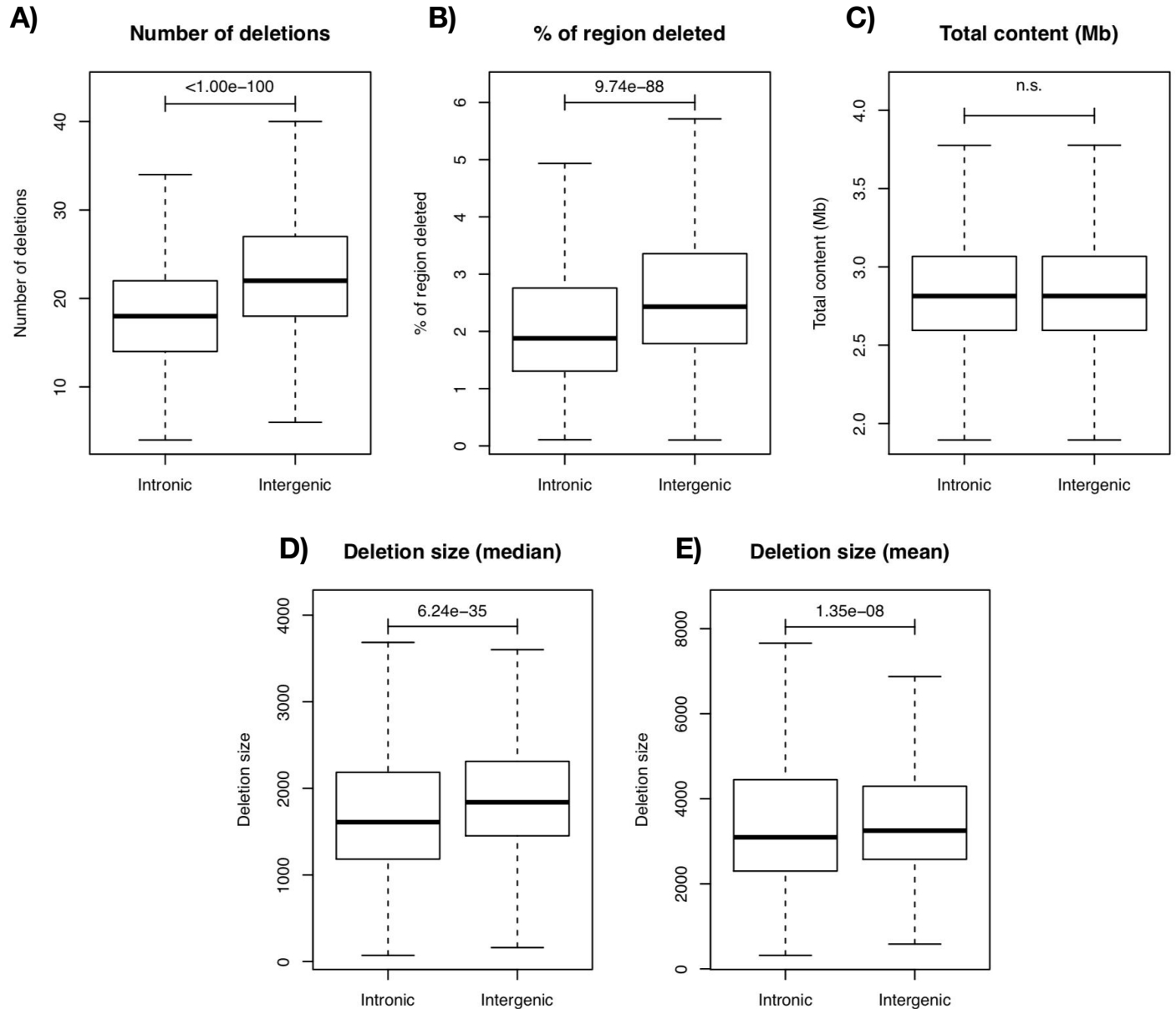

Supplement: S4 Fig — Groups of 500 introns were randomly sampled 10,000 times and paired each time with 500 intergenic regions of similar size. In every permutation, their deletion content was compared. Each point in a boxplot corresponds to a single value per randomization. A) Total number of deletions in 500 intronic or intergenic regions. B) Percentage of the sequence that is lost in each subset of 500 intronic/intergenic regions. C) Total amount of genome comprised in each subset, to verify that the intronic and intergenic subsets they have similar sizes (no significant differences). D) Median and E) mean size of deletions within intronic and intergenic regions. P-values were calculated with paired Student’s T-test comparisons. (PDF) [file pgen.1007902.s004.pdf]

## Sudmant (Nature)

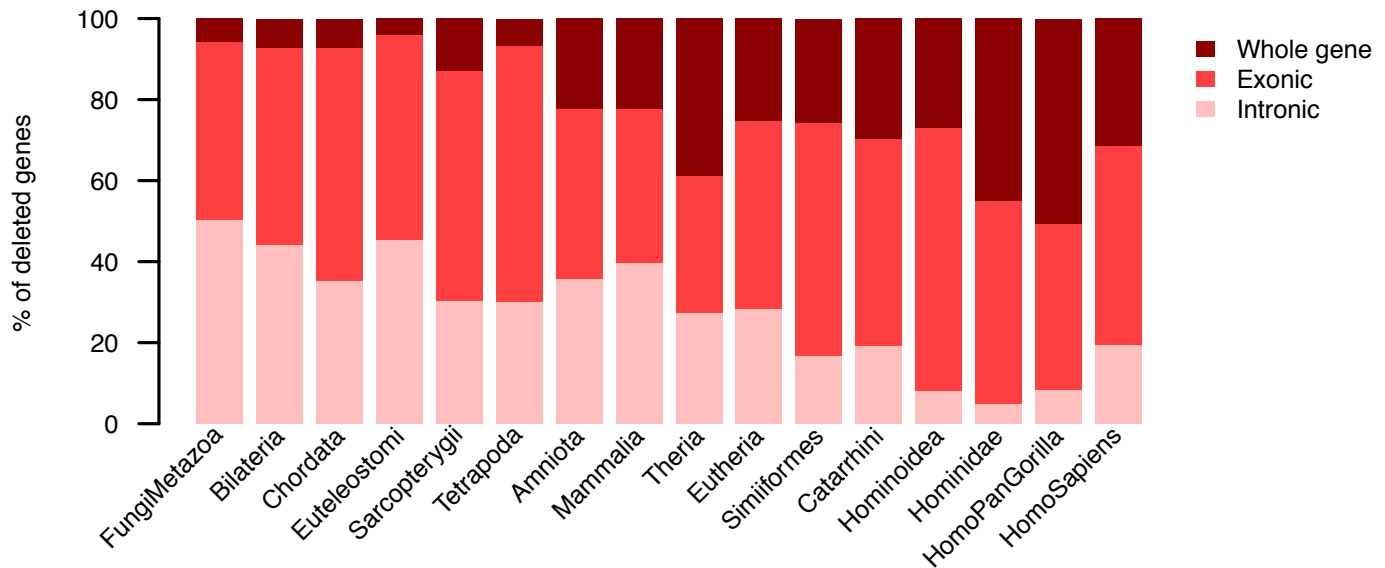

## Zarrei

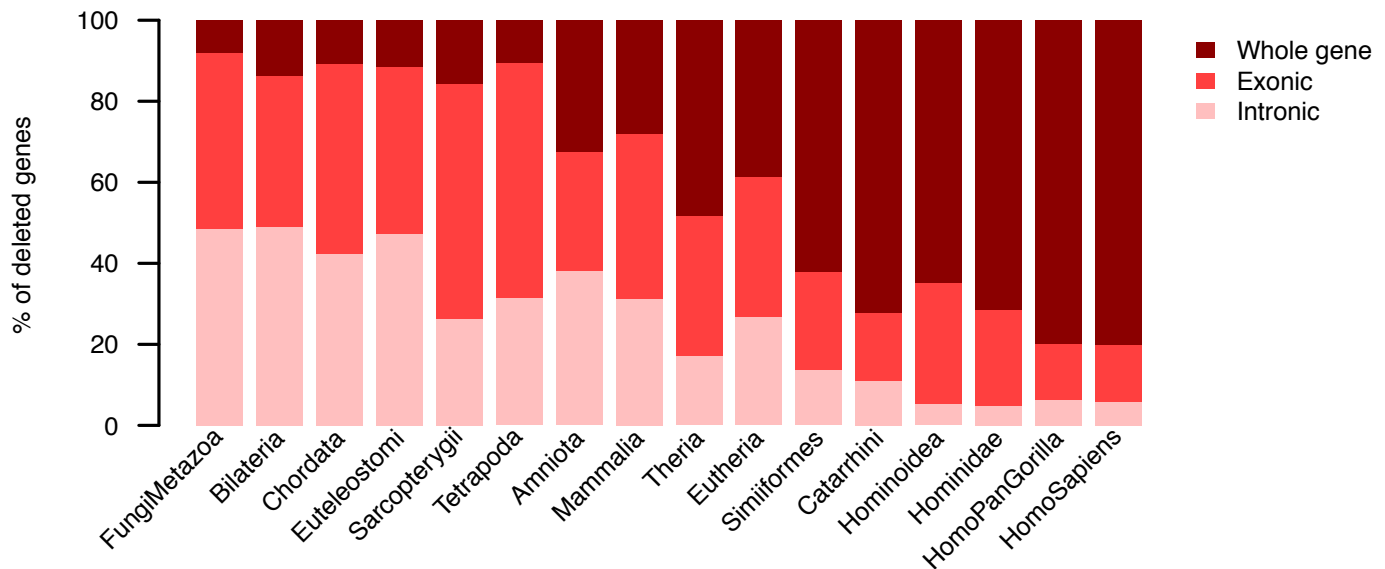

## Abyzov

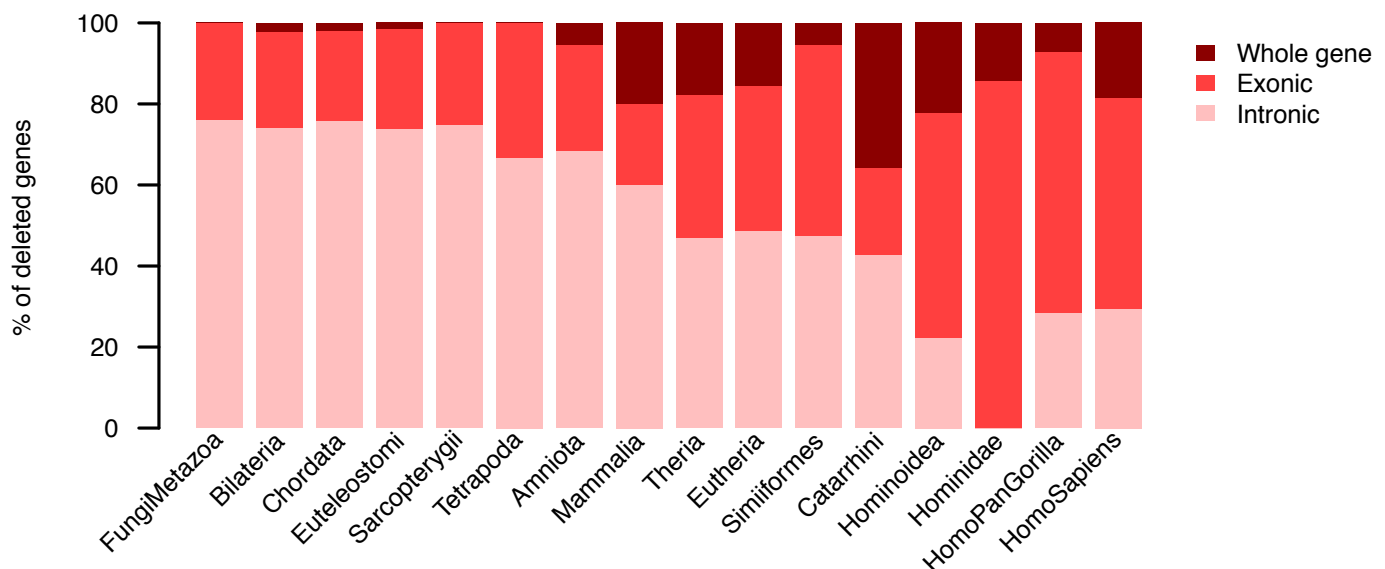

Supplement: S8 Fig — Proportion of genes with deletions that have the whole locus deleted, only part of their exons (exonic) affected by deletions or intronic deletions only. This figure is equivalent to Fig 3C, but here separated by CNV map. (PDF) [file pgen.1007902.s008.pdf]

**A)**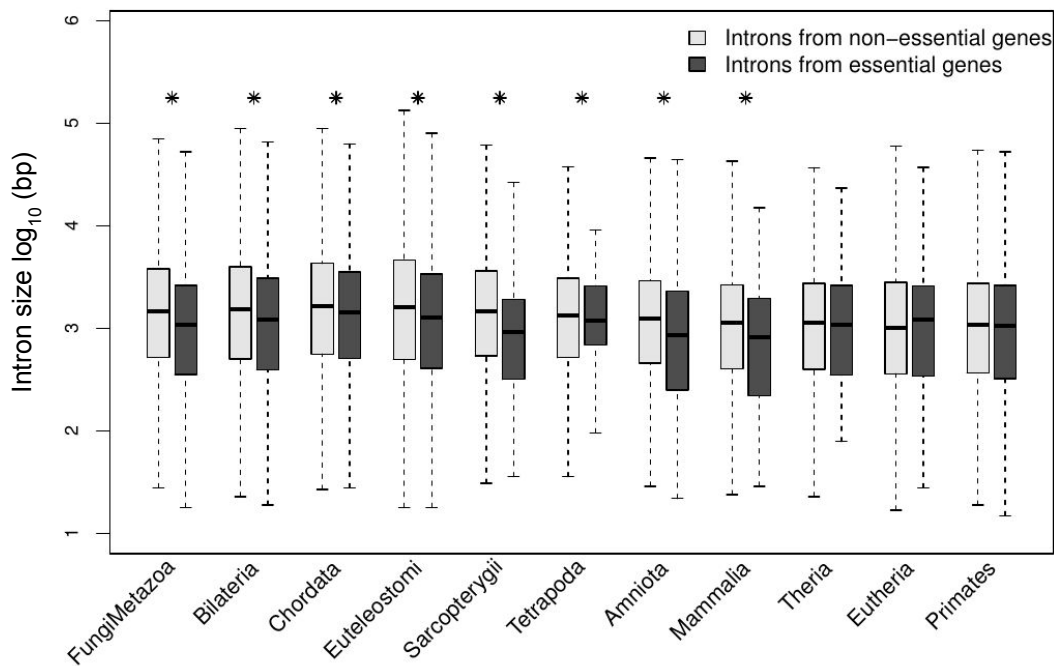**B)**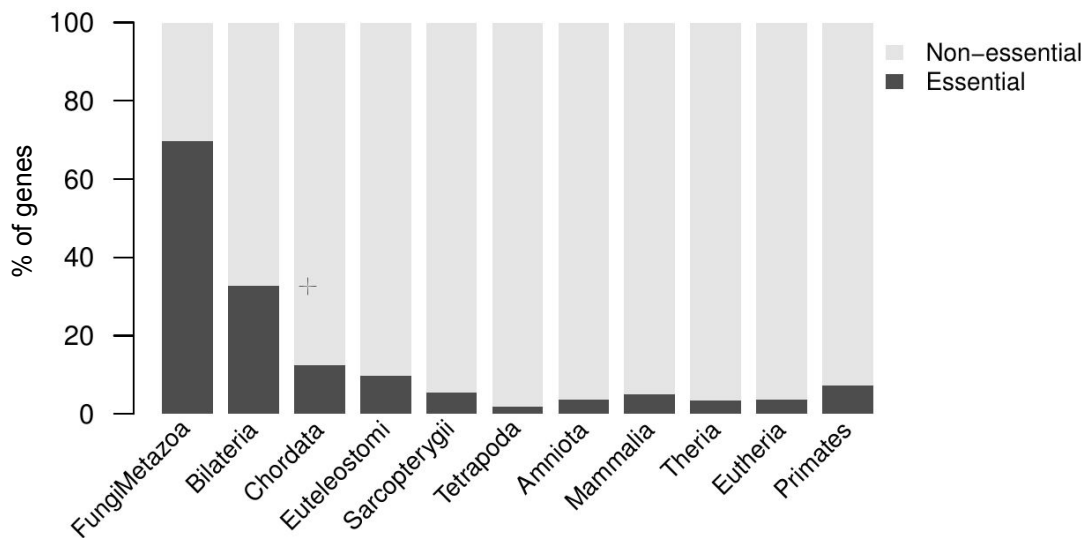

Supplement: S9 Fig — (A) Intron sizes of non-essential and essential genes. (B) Percentage of essential genes per evolutionary age. (PDF) [file pgen.1007902.s009.pdf]

## A) GC difference (Exon – intron)

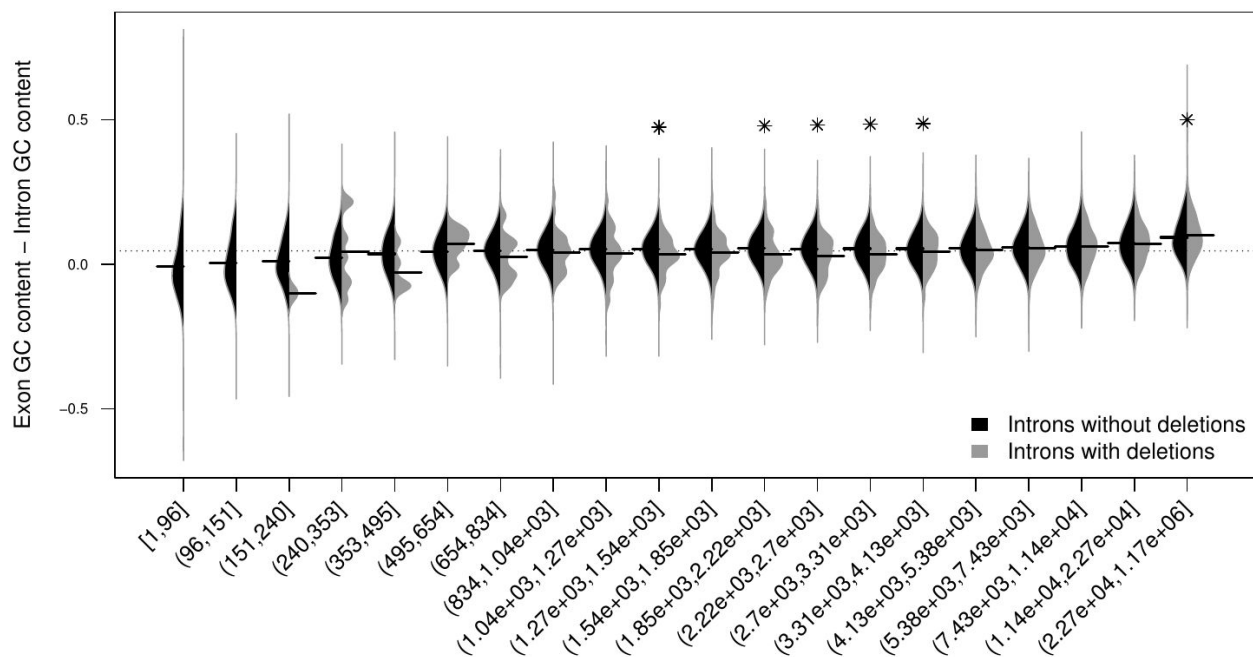

## B) GC content in introns

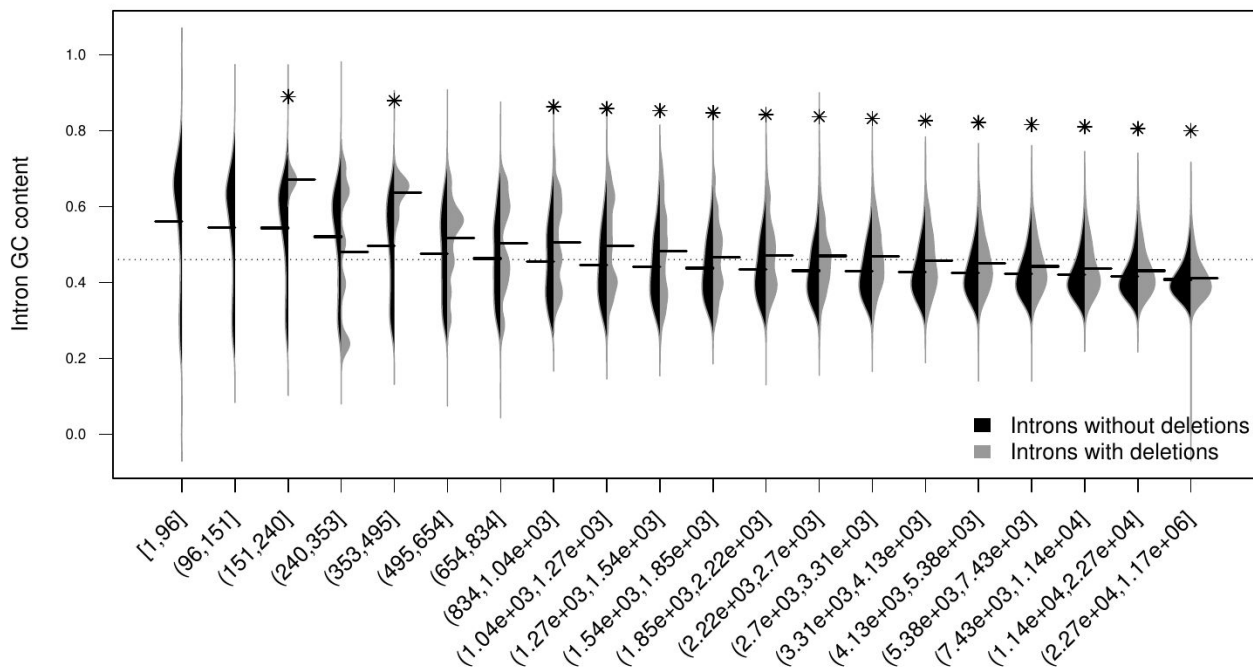

Supplement: S11 Fig — (A) Bean-plots showing the different GC distribution between the flanking exons of introns with or without deletions, separated by intron size bins (with equal number of introns per bin). (B) GC content distributions in introns with or without deletions, separated by intron size bins. Significance is considered for p-values < 0.05. Beans show the estimated density of each distribution; horizontal lines show the mean values of each side of the bean and the dashed horizontal line line represents the average of all values. (PDF) [file pgen.1007902.s011.pdf]
