# Supplementary material for: Intronic CNVs and gene expression variation in human populations
Source: PLoS Genet. 2019 Jan 24;15(1):e1007902. doi: 10.1371/journal.pgen.1007902 (PMC6345438; doi:10.1371/journal.pgen.1007902)

# Enrichment of number of deletions in introns of different sizes

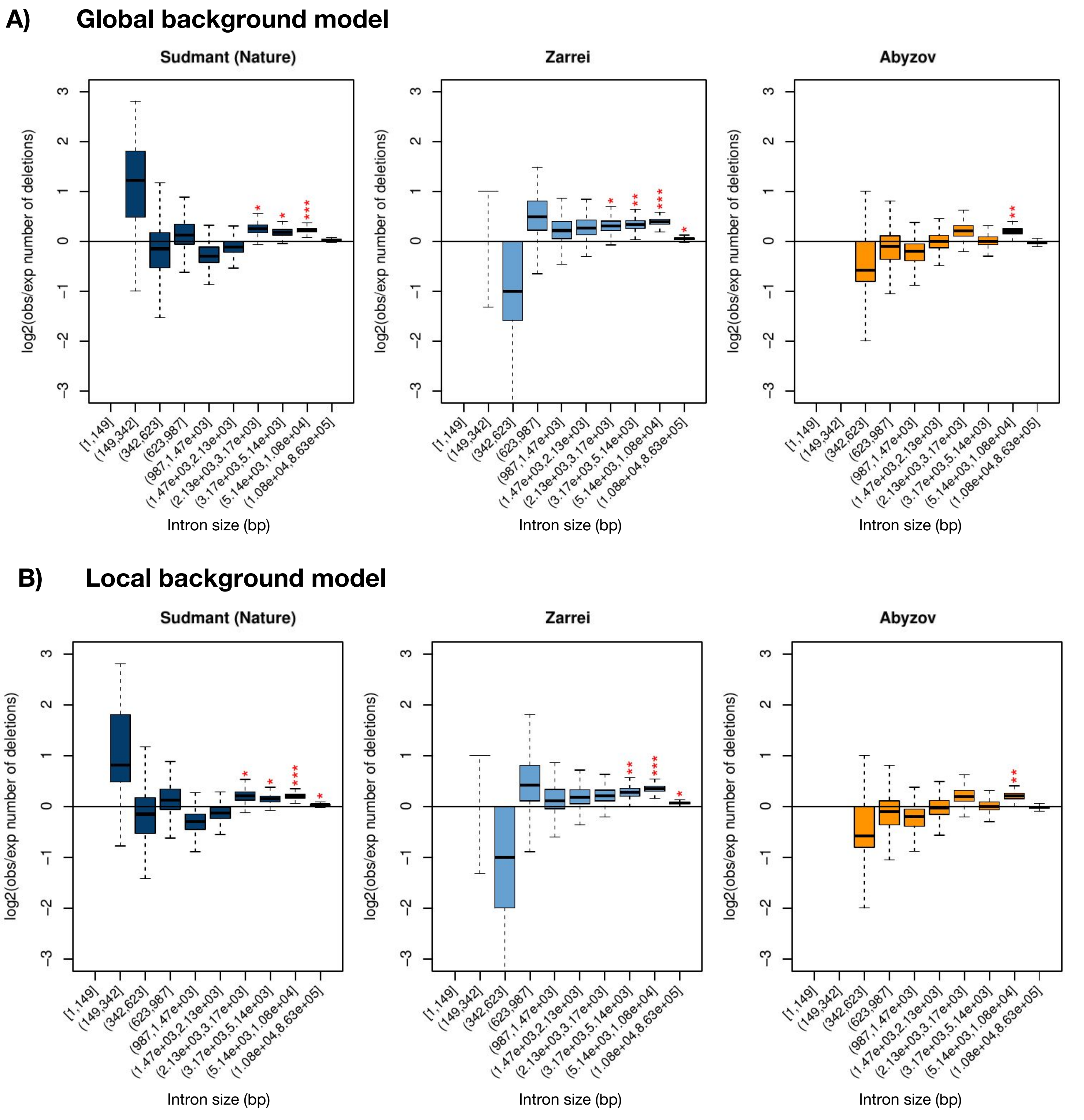

Supplement: S3 Fig — Ratios of observed versus expected number of deletions in each size bin after 10,000 random permutations using global (A) and local (B) background models. All size bins have a similar number of intronic regions (deciles, size intervals indicated between brackets). Asterisks mark the bins significantly enriched with intronic deletions: * for P<0.05, ** for P<0.005 and *** for P<0.0005. (PDF) [file pgen.1007902.s003.pdf]
