# Supplementary material for: Intronic CNVs and gene expression variation in human populations
Source: PLoS Genet. 2019 Jan 24;15(1):e1007902. doi: 10.1371/journal.pgen.1007902 (PMC6345438; doi:10.1371/journal.pgen.1007902)

# Enrichment of CNVs (including gains) in genes of different evolutionary ages

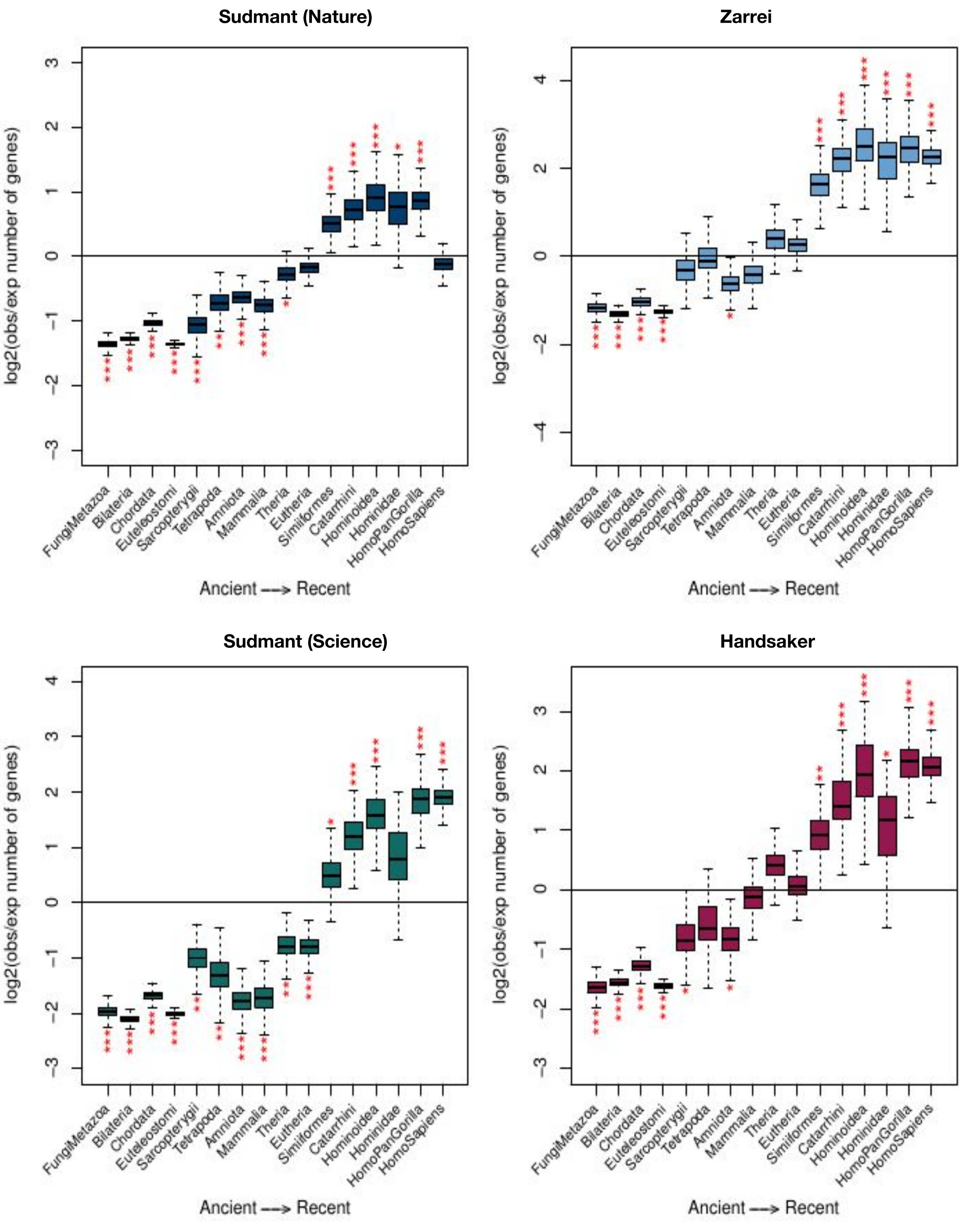

Supplement: S5 Fig — Ratios of observed versus expected number of genes with CNVs (gains, losses and gain and loss CNVs) affecting their coding region in each gene age after 10,000 random permutations using the global background model. Abyzov’s map [17] is excluded because it is the only CNV map that does not contain any gain. Red asterisks show an enrichment when above the box, a depletion when below the box: * for P<0.05, ** for P<0.005 and *** for P<0.0005. (PDF) [file pgen.1007902.s005.pdf]
