# Supplementary material for: Intronic CNVs and gene expression variation in human populations
Source: PLoS Genet. 2019 Jan 24;15(1):e1007902. doi: 10.1371/journal.pgen.1007902 (PMC6345438; doi:10.1371/journal.pgen.1007902)

Percentage of genes with purely intronic or coding deletions

A) Intronic deletions

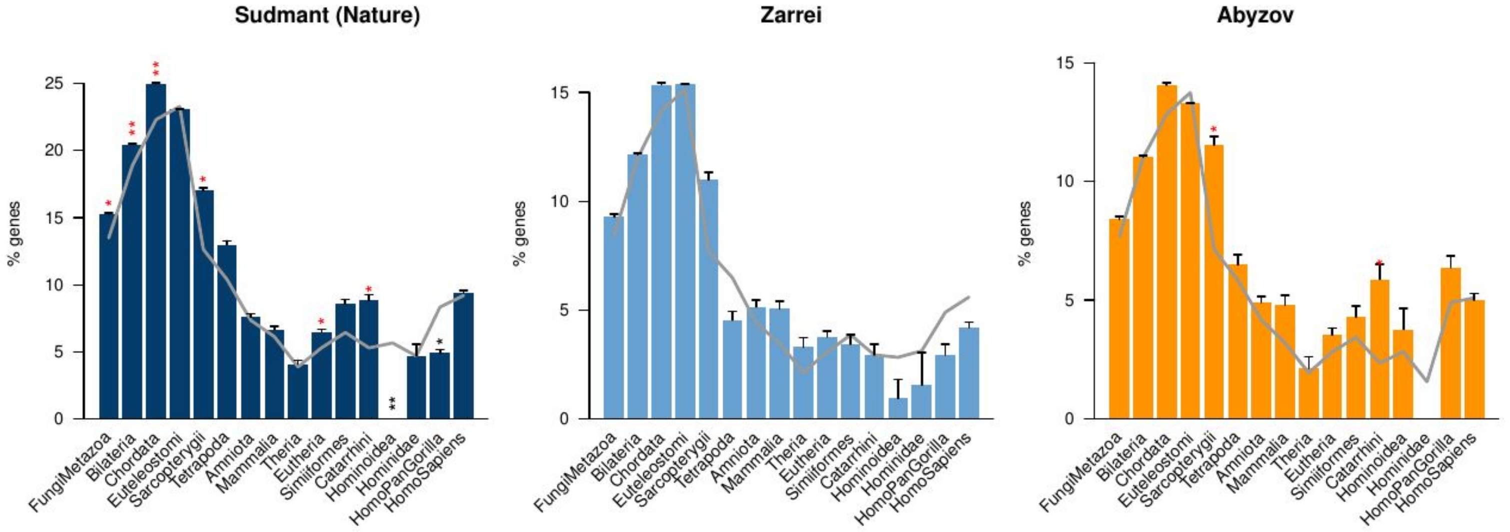

B) Coding deletions

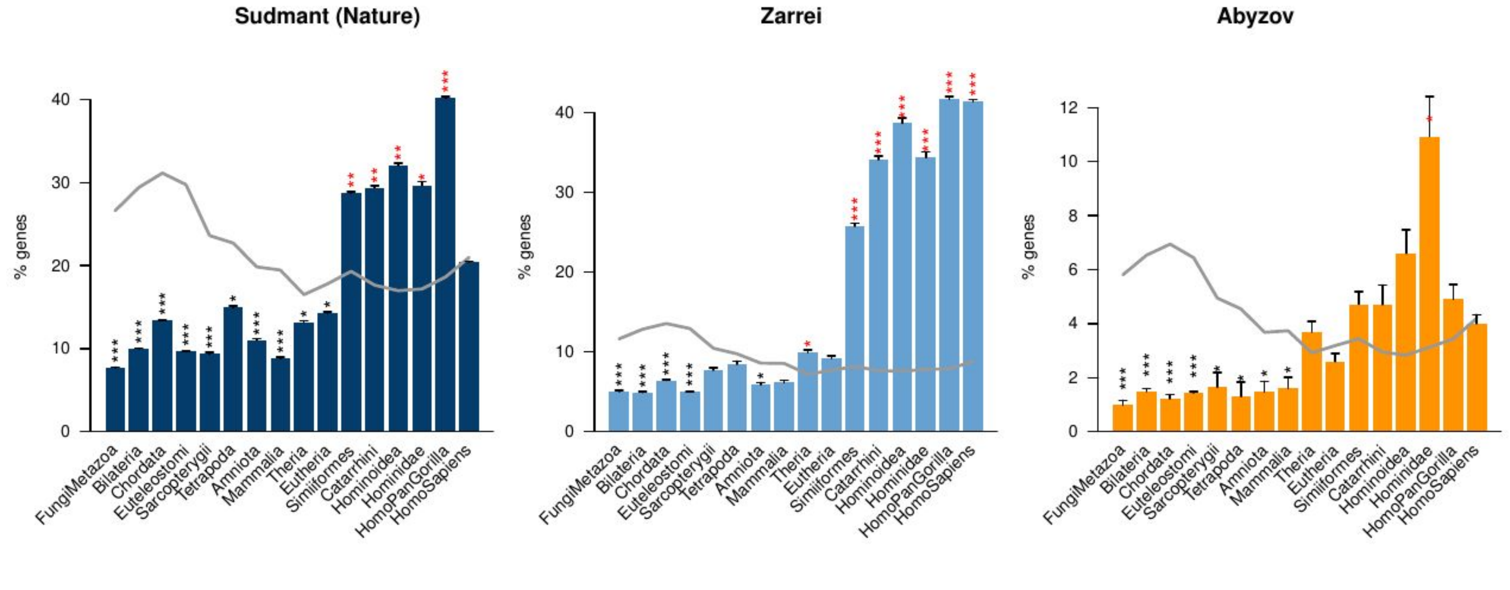

Supplement: S6 Fig — Percentage of genes from each gene evolutionary age that contain intronic deletions in (A) or deletions overlapping with exons (including partial and whole gene CNVs) in (B). The gray line represents the expected value, calculated as the median of the genes in the 10,000 random permutations. Significance is marked with asterisks: * for P<0.05. ** for P<0.005.*** for P<0.0005 and their color represents enrichment (red) or impoverishment (black). (PDF) [file pgen.1007902.s006.pdf]
