# Supplementary material for: Intronic CNVs and gene expression variation in human populations
Source: PLoS Genet. 2019 Jan 24;15(1):e1007902. doi: 10.1371/journal.pgen.1007902 (PMC6345438; doi:10.1371/journal.pgen.1007902)

Enrichment of number of deletions in introns bigger than 1500 bp

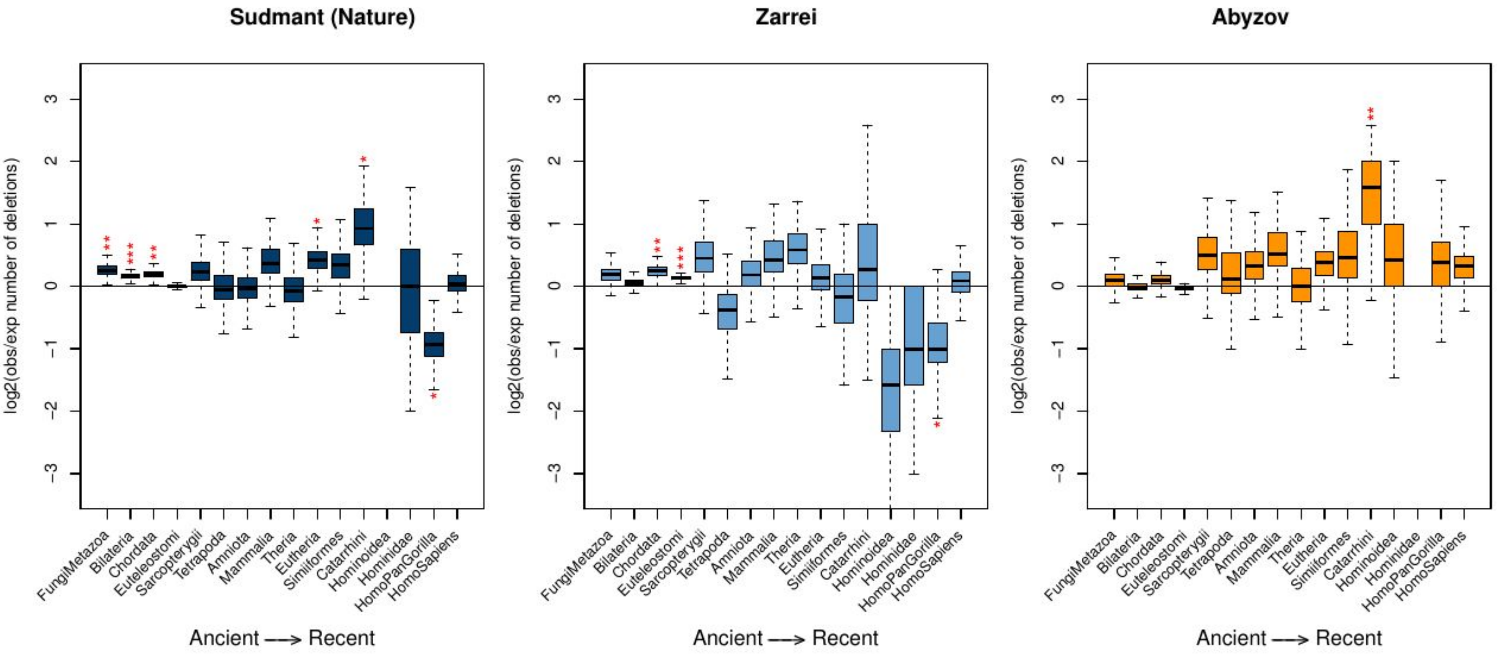

Supplement: S7 Fig — Ratios of observed versus expected number of deletions within introns bigger than 1.5kb from different evolutionary ages. Expected values are calculated 10,000 random permutations using a global background model. Asterisks show an enrichment when above the box, a depletion when below the box: * for P<0.05, ** for P<0.005 and *** for P<0.0005. (PDF) [file pgen.1007902.s007.pdf]
