# Supplementary material for: Intronic CNVs and gene expression variation in human populations
Source: PLoS Genet. 2019 Jan 24;15(1):e1007902. doi: 10.1371/journal.pgen.1007902 (PMC6345438; doi:10.1371/journal.pgen.1007902)

Stratified intronic eDeletions

A) Characteristics of the intronic eDeletions and the gene that carries them

Gene	eDeletion	Allele freq.	Type	FCs	Adjusted P-value	max V_{st}	Gene age	RVIS
EXOC2	chr6:666377-667821	0.5651	intronic (*cis*)	-1.1097	0.00028	0.307283	Bilateria	3.34
SKAP2	chr7:26877406-26877456	0.0839	intronic (*cis*)	-1.2070	0.00933	0.323179	Euteleostomi	64.26
PTGR1	chr9:114333161-114333923	0.0982	intronic (*cis*)	-1.3235	0.01555	0.280086	Bilateria	54.73
PHYHD1	chr9:131700957-131702150	0.2596	intronic (*cis*)	-1.9754	0.02005	0.251624	Bilateria	87.20

B) Gene expression in individuals carrying or not the deletion

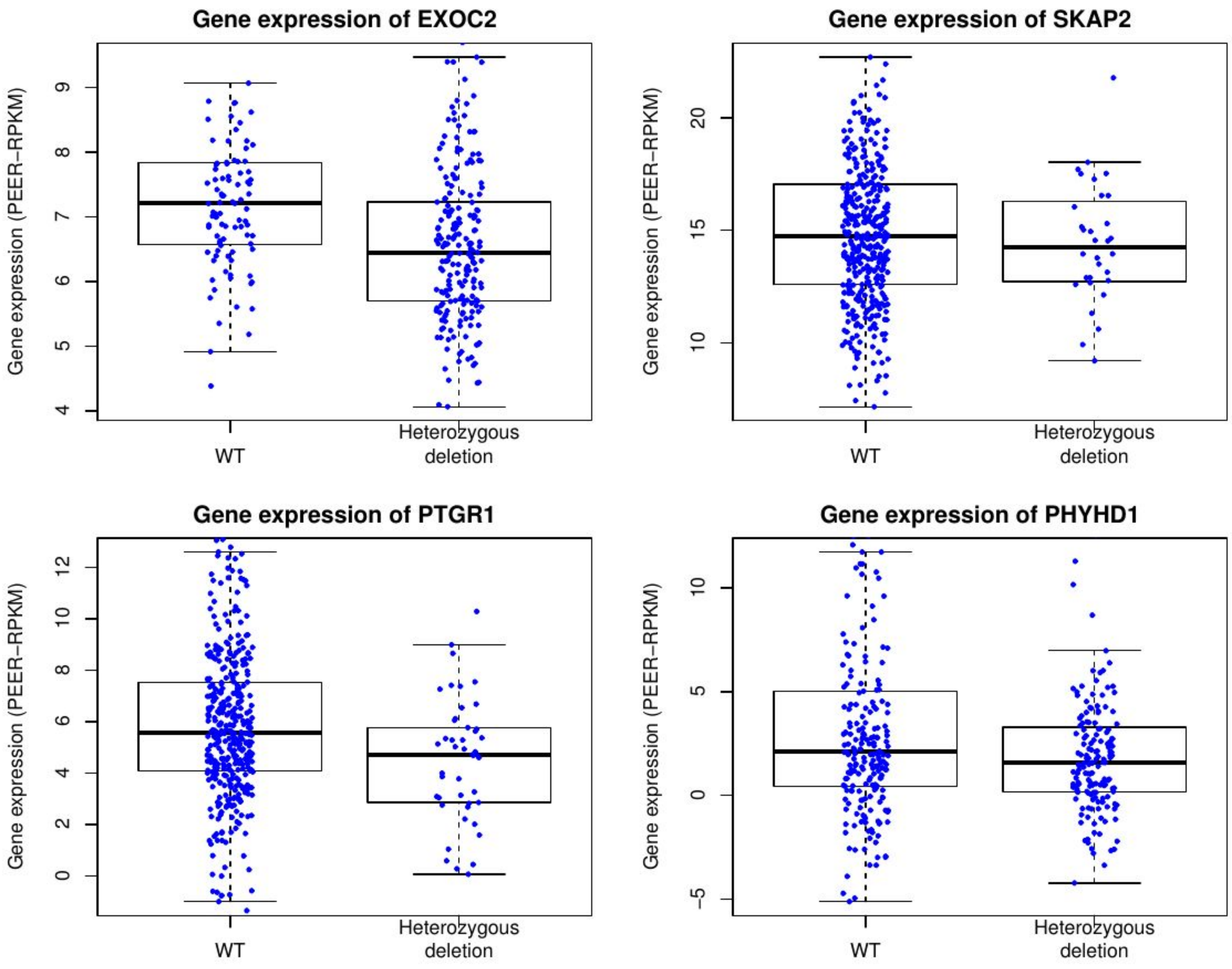

Supplement: S10 Fig — (A) Characteristics of highly stratified variants (HSVs) that are significant cis-intronic-eDeletions. (B) Gene expression of “wild-type” (CN = 2) individuals and heterozygous carriers (CN = 1) of the eDeletion. (PDF) [file pgen.1007902.s010.pdf]
