# Supplementary material for: Intronic CNVs and gene expression variation in human populations
Source: PLoS Genet. 2019 Jan 24;15(1):e1007902. doi: 10.1371/journal.pgen.1007902 (PMC6345438; doi:10.1371/journal.pgen.1007902)
Supplement: S12 Fig — X-axis represents the coordinates of the intron with its flanking exons (black boxes). Y-axis shows the GC content, calculated with sliding 200bp windows. The deleted region is highlighted in grey. (PDF) [file pgen.1007902.s012.pdf]

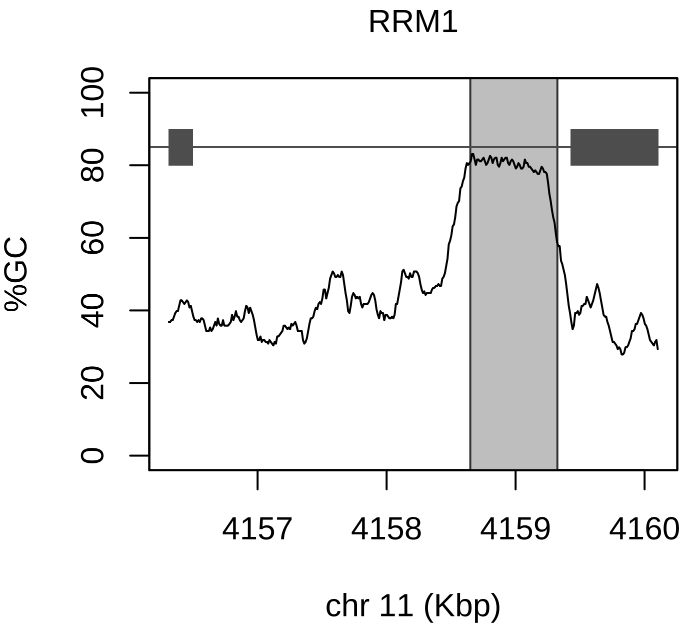

Gene size reduction: 1.5%

GC before deletion: 0.51

GC after deletion: 0.43

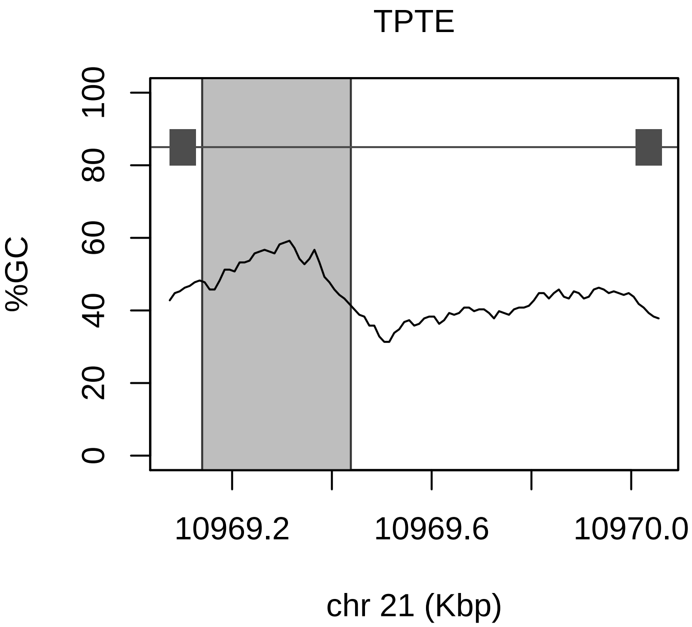

Gene size reduction: 0.4%

GC before deletion: 0.44

GC after deletion: 0.4
