# Supplementary material for: Intronic CNVs and gene expression variation in human populations
Source: PLoS Genet. 2019 Jan 24;15(1):e1007902. doi: 10.1371/journal.pgen.1007902 (PMC6345438; doi:10.1371/journal.pgen.1007902)

Alu element content in deletions from Abyzov et al. 2015

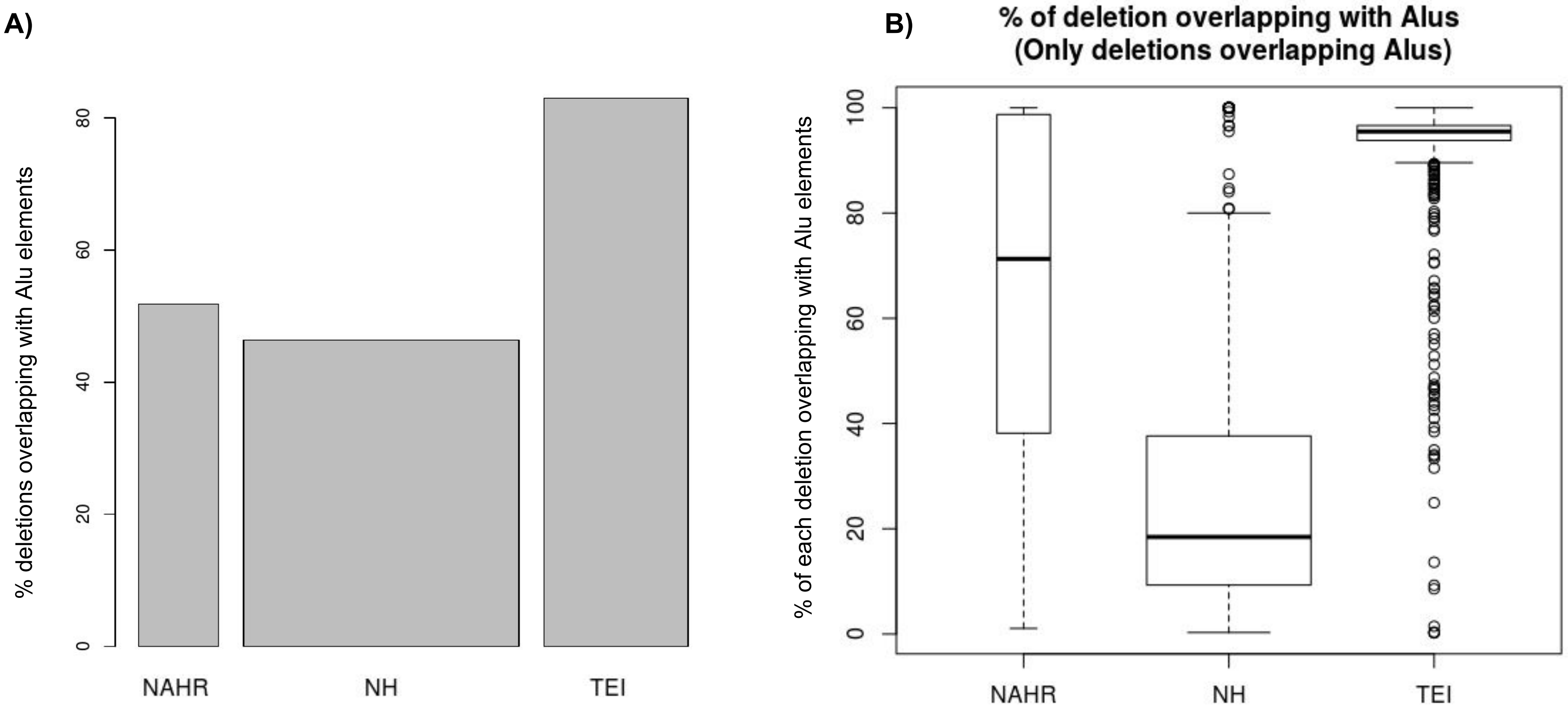

Supplement: S13 Fig — (A) Proportion of deletions of each mechanism that overlaps with Alu elements. (B) Percentage of the deleted regions covered by Alu elements. Deleted regions and mechanisms from Abyzov’s map [17]. NAHR: Non-allelic homologous recombination. NH: Non-homologous end joining. TEI: Transposable Element Insertion. (PDF) [file pgen.1007902.s013.pdf]
