# Supplementary material for: Intronic CNVs and gene expression variation in human populations
Source: PLoS Genet. 2019 Jan 24;15(1):e1007902. doi: 10.1371/journal.pgen.1007902 (PMC6345438; doi:10.1371/journal.pgen.1007902)
Supplement: S1 Table — (PDF) [file pgen.1007902.s014.pdf]

Number of individuals in each map, project the variants belong to and methods used for CNV detection.

Map	Abyzov	Handsaker	Zarrei	Sudmant (Science)	Sudmant (Nature)
Individuals	1092	849	2647	236	2504 (from 26 populations)
Project	1000 Genomes Project, Phase 1	1000 Genomes Project, Phase 1	Meta analysis of DGV collection		1000 Genomes Project, Phase 3
Methods	WGS, Split read	WGS, Read -depth	Multiple genome-wide techniques	WGS, Read-depth	WGS, Multiple algorithms
