# Supplementary material for: Intronic CNVs and gene expression variation in human populations
Source: PLoS Genet. 2019 Jan 24;15(1):e1007902. doi: 10.1371/journal.pgen.1007902 (PMC6345438; doi:10.1371/journal.pgen.1007902)
Supplement: S5 Table — (PDF) [file pgen.1007902.s018.pdf]

	Whole gene	Exonic	Intronic - cis	Intronic - trans	Intergenic
Number of eTranscripts (number eDeletions)	22*** (11)	135*** (92)	217* (199)	81 (54)	123 (96)
Expected number of eTranscripts (median ± median absolute dev.)	4 ± 1.48	67 ± 10.38	173 ± 19.27	75 ± 10.38	109 ± 13.34
Number of genes ≥ 1 eTranscript	11 **	87 ***	185 **	65	104
Expected number of genes with ≥1 eTranscript (median ± median absolute dev.)	4 ± 1.48	53 + 7.41	143 ± 14.83	64 ± 8.90	94 ± 10.38
Proportion of downregulated eTranscripts	100%	91%	79%	81%	89%
Total genes tested for transcript differential expression (total deletions tested)	47 (43)	403 (440)	1401 (1886)	653 (319)	972 (529)
