# Supplementary material for: Intronic CNVs and gene expression variation in human populations
Source: PLoS Genet. 2019 Jan 24;15(1):e1007902. doi: 10.1371/journal.pgen.1007902 (PMC6345438; doi:10.1371/journal.pgen.1007902)
Supplement: S6 Table — (PDF) [file pgen.1007902.s019.pdf]

Overlap of intronic deletions with regulatory features.

**$\log_2$ Fold Change (p-value)**	Sudmant (Nature)	Zarrei	Abyzov
CTCF binding site	**-0.18 (0.0072)**	-0.14 (0.11018)	**-0.81 (0.0001)**
Enhancer	**-0. 14 (0.0304)**	**-0.45 (0.0006)**	**-0.47 (0.0045)**
