# Supplementary material for: Intronic CNVs and gene expression variation in human populations
Source: PLoS Genet. 2019 Jan 24;15(1):e1007902. doi: 10.1371/journal.pgen.1007902 (PMC6345438; doi:10.1371/journal.pgen.1007902)
Supplement: S1 Appendix — (DOCX) [file pgen.1007902.s022.docx]

**S1 Appendix**

**CNV gains and losses relative to the human reference genome**

The methods originally applied to generate the five CNV maps included in our study use the sequence in the reference human genome as a baseline. Each individual genome is aligned to the reference and CNVs are generally called as regions that are “gained” or “lost” in that individual genome. Gains are generally assumed to be extra copies of the CNV regions (either in tandem or somewhere else in the personal genome) while losses are assumed to be homozygous or heterozygous deletions.

In our work we have focused in CNV losses that only affect intronic sequence, and we refer to these events as either intronic deletions or intronic losses (because some individuals in the population lack this regions). However, it is worth noting that although they are deletions relative to the reference genome, the mutations responsible may have originally been insertions or deletions.

To unravel the ancestral state of the CNVs marked as deletions, we have compared the Final 1000 Genomes Project dataset (Sudmant Nature) with recent high-quality genomic data of great apes released during the revision process of our paper (Kronenberg et al. 2018, ref. 23). In detail, when comparing Sudmant (Nature) to Kronenberg et al. (2018), an SV was considered identical if there was a reciprocal overlap higher than 80%. Deletions were confirmed when they appeared in a genomic region that can be found in non-human primates (NHP), without any SV in the NHP or with insertions only. Conversely, insertions were confirmed if the fragment is annotated as a deletion in all NHP at an allelic frequency = 1.

We were able to confirm that at least 72.8% (16319/22412) of the deletions are actual deletions (79.2% of all intronic deletions), compared to the ancestral state. On the other hand, we observed that 0.3% (70/22412) of the deletions are insertions (0.42% of the intronic deletions). For the remaining ~27% of deletions it is difficult to assign the ancestral state, in some cases because we would need more species to compare and in others because we find structural variants (SVs) different to those observed in human populations, pointing to a more complex scenarios that could involve more than one event. Consequently, we found that most CNV losses correspond to deletions in human individuals and that they should be regarded as such when interpreting their presence or absence in different genomic regions.

Reference 23: [Kronenberg ZN, Fiddes IT, Gordon D, Murali S, Cantsilieris S, Meyerson OS, et al. High-resolution comparative analysis of great ape genomes. Science. 2018;360. doi:](http://paperpile.com/b/egqrMr/yptk)[10.1126/science.aar6343](http://dx.doi.org/10.1126/science.aar6343)
